# Estimating the Basic Reproductive Number (*R_0_*) for African Swine Fever Virus (ASFV) Transmission between Pig Herds in Uganda

**DOI:** 10.1371/journal.pone.0125842

**Published:** 2015-05-04

**Authors:** Mike B. Barongo, Karl Ståhl, Bernard Bett, Richard P. Bishop, Eric M. Fèvre, Tony Aliro, Edward Okoth, Charles Masembe, Darryn Knobel, Amos Ssematimba

**Affiliations:** 1 Department of Academic Registrar (ICT Division), Makerere University, Kampala, Uganda; 2 International Livestock Research Institute, Nairobi, Kenya; 3 Department of Disease Control and Epidemiology, National Veterinary Institute, Uppsala, Sweden; 4 Ministry of Agriculture, Animal Industry and Fisheries, Entebbe, Uganda; 5 Department of Biological Sciences, College of Natural and applied Sciences, Makerere University, Kampala, Uganda; 6 Department of Veterinary Tropical Diseases, Faculty of Veterinary Science, University of Pretoria, Pretoria, South Africa; 7 Department of Mathematics, Faculty of Science, Gulu University, Gulu, Uganda; Virginia Polytechnic Institute and State University, UNITED STATES

## Abstract

African swine fever (ASF) is a highly contagious, lethal and economically devastating haemorrhagic disease of domestic pigs. Insights into the dynamics and scale of virus transmission can be obtained from estimates of the basic reproduction number (*R*_0_). We estimate *R*_0_ for ASF virus in small holder, free-range pig production system in Gulu, Uganda. The estimation was based on data collected from outbreaks that affected 43 villages (out of the 289 villages with an overall pig population of 26,570) between April 2010 and November 2011. A total of 211 outbreaks met the criteria for inclusion in the study. Three methods were used, specifically; (i) GIS- based identification of the nearest infectious neighbour based on the Euclidean distance between outbreaks, (ii) epidemic doubling time, and (iii) a compartmental susceptible-infectious (SI) model. For implementation of the SI model, three approaches were used namely; curve fitting (CF), a linear regression model (LRM) and the *SI/N* proportion. The *R*_0_ estimates from the nearest infectious neighbour and epidemic doubling time methods were 3.24 and 1.63 respectively. Estimates from the SI-based method were 1.58 for the CF approach, 1.90 for the LRM, and 1.77 for the *SI/N* proportion. Since all these values were above one, they predict the observed persistence of the virus in the population. We hypothesize that the observed variation in the estimates is a consequence of the data used. Higher resolution and temporally better defined data would likely reduce this variation. This is the first estimate of *R_0_* for ASFV in a free range smallholder pig keeping system in sub-Saharan Africa and highlights the requirement for more efficient application of available disease control measures.

## Introduction

African Swine Fever (ASF) is a highly contagious, lethal and economically devastating haemorrhagic fever of domestic pigs. The disease is of high economic importance both globally and in sub-Saharan Africa where demand for animal protein including pork has greatly increased in the last two decades [1,2].

The disease is caused by African Swine Fever virus (ASFV), a large double-stranded DNA-virus and sole member of the family *Asfarviridae* [3]. ASFV isolates vary in their virulence, from highly virulent isolates that kill up to 100% of the pigs to moderately or low virulence viruses with mortalities ranging between 30–70% [4,5]. ASF produces clinical signs that range from peracute, acute, sub-acute and chronic forms depending on the virulence of the strain, intensity of exposure and pig breed [6,7]. The disease is characterised by high fever, loss of appetite, haemorrhages in the skin and internal organs, and death. Pigs that apparently recover from the disease become virus carriers [5].

ASF has spread and is now established in many sub-Saharan countries since its discovery in Kenya in 1921 [8]. Initially it was reported from countries in East and Southern Africa but has now spread through Central and West Africa, and Indian Ocean islands with Chad becoming the most recent country to be affected [9]. The disease first spread outside the African continent to Portugal in 1957. From 1968–1995, ASFV in the p72 genotype I was present in European countries including Malta, Sardinia, Italy, France, Belgium and the Netherlands. The prevalent genotype in Gulu district is genotype IX. It was eradicated in all these countries except Sardinia where it remains endemic and poses a continuous risk of re-introduction and spread in Europe [10,11]. ASF was accidentally introduced into the Caucasus in 2007 from where it spread rapidly and widely within the Russian Federation. Outbreaks were reported more recently in Poland, Lithuania, Latvia and Estonia [12,13].

This virus is stable at a wide range of temperatures and pH and is capable of remaining infective in faeces, tissue and environment for many days [10]. The incubation period in domestic pigs varies from 5 to 15 days depending on the virus genotype [5]. ASFV is maintained in two main cycles: a sylvatic cycle that involves natural hosts, namely warthogs and soft ticks (*Ornithodoros moubata*) and a domestic cycle that may not involve the soft ticks [14]. In the domestic cycle, ASFV can be transmitted by direct contact with infected animals, indirect contact through fomites, and tick vectors. Transmission in the domestic cycle is exacerbated by sociocultural factors such as pig movement networks (traders, butchers, boar service), superstition and beliefs (e.g. that a carcass cannot be buried), use of untreated swill, lack of confinement of pigs and low biosecurity adoption [15,16].

There is currently no available vaccine against ASF and the available control strategies focus on preventing and controlling the spread of the virus although in better-resourced parts of the world, “stamping out” of pigs within infected farms and surrounding areas is used [10,17].

This study was based on data from confirmed outbreaks that occurred in Gulu district, northern Uganda, in the period 2010–2011. The main economic activity in the district is subsistence agriculture which engages up to 90% of the population, with 9% of the households involved in pig farming. The pig production systems practiced in the study area are predominantly traditional free ranging and tethering, supplemented by very limited semi-intensive and intensive farming with virtually no biosecurity measures in regular use. In addition to the roaming of free ranging pigs, movements in the area occur for purposes of restocking, breeding, and trading [2,16]. Moving of apparently asymptomatic pigs to neighbouring villages when an outbreak is suspected is an additional factor that may promote virus transmission.

Estimates of the basic reproductive number (*R*_*0*_) are fundamental in underpinning rational control strategies based on disease modelling. *R*_*0*_ is the average number of secondary cases arising from a single infectious individual in a wholly susceptible population throughout its infectious period [18–23]. This parameter can be estimated using a variety of mathematical techniques [17,20,24]. This estimate provides a means to better understand the dynamics of infectious disease outbreaks and to assess the potential efficacy of disease control measures [25]. It is frequently used as a threshold parameter to quantify the spread of disease and is therefore a quantitative indicator of both the risk of an epidemic and the effort required to control it in a particular population. In order to control an infectious disease, it is necessary to reduce *R*_*0*_ to below unity (S1 Text) [25,26]. This parameter can predict the speed and scale of disease spread and the level of herd immunity required to contain the disease [17].

In many resources-constrained small holder communities, such as in East Africa, information on transmissibility of diseases like ASF is often lacking and usually limited to daily counts of new cases [27,28]. Additionally, decisions about the best control strategies to implement during an epidemic are complex, usually involving technical, political, sociocultural and economic issues. ASF outbreaks are not reported quickly enough to allow collection of all the required empirical data for the estimation of disease parameters. To overcome this constraint, indirect methods can be used to estimate these parameters, including *R*_*0*_. There are a number of approaches available for estimating *R*_*0*_. Some methods are purely analytical, and not very reliable [29], while others are mathematical expressions involving multiple population parameters that have to be estimated separately [25,30] using outbreak data [20,24,31]. Ward et al. [20], Bett et al. [24] and Li et al. [29] describe different methods for estimating *R*_*0*_ from outbreak data for a number of diseases across different geographical regions. In this study, we estimate *R*_*0*_ for ASF transmission between herds of pigs based on data from confirmed outbreaks using some of these methods as described next.

## Materials and Methods

This is to certify that any sampling of live or dead pigs described within the manuscript PONE-D-14-46309 Estimating the basic reproductive number (*R*_*0*_) for African swine fever virus (ASFV) transmission between pig herds in Uganda by Barongo et al, was conducted in close collaboration between the District veterinary office in Gulu District and scientists from Uganda, Kenya and Sweden as part of disease investigations for African swine fever, and funded through a collaborative research project. Disease surveillance and disease investigations lie within the mandate of the District veterinary office. The district veterinarian thus holds a general permission to sample animals for this purpose. Data from these disease investigations were reported to the National Animal Disease Diagnostic and Epidemiology Centre, NADDEC, under the ministry of Agriculture Animal Industry and Fisheries in Entebbe. The data also formed part of the basis for international reporting to the OIE. No samples were collected specifically for the manuscript submitted to PLOS ONE.

### Data source

We used data collected during previous research activities from villages in Gulu District with laboratory confirmed outbreaks of ASF (material described in [32,33]). Outbreaks included in this study occurred between April 2010 and November 2011. In brief, villages that reported outbreaks of disease characterized by fever and mortality in pigs to the district veterinary authorities were visited. Within each village, samples (blood and serum) were collected from clinically diseased and/or apparently healthy pigs from all affected households. Samples were kept cool awaiting transportation to the Molecular Biology Laboratory at Makerere University Institute of Environment and Natural Resources (MUIENR) in Kampala for storage at -20°C until further processing. In the laboratory, outbreaks were confirmed by detection of ASFV nucleic acids using a commercially available real-time PCR (Tetracore Inc., Rockville, Maryland) in accordance with the instructions of the manufacturer [34]. During a second visit to all villages with laboratory confirmed outbreaks, additional data was collected using semi-structured questionnaires from a total of 211 households. The data collected included farm location (GPS coordinates), start month of the confirmed outbreak, number of pigs that had died, number that were still alive, disposal mechanism of carcasses, feed source and production system practiced.

A herd, here defined as a collection of all pigs in a pig-keeping household, was taken to be the epidemiological unit of interest [20]. Thus, our estimates of *R*_*0*_ reflect spread between herds. All outbreaks in the district during the period of study are assumed to have been reported. Additionally, it was assumed that all herds in the district were susceptible during the study period and the pigs from different herds were homogeneously mixing [2]. The exact number of herds present during the period of the study could not be directly determined and we estimated from the National Livestock Census Report (2008) on distribution of livestock in Uganda that there were 6,200 pig herds (mean herd size of 4.3) distributed over the 289 villages in Gulu district.

### Data Analysis

Three methods, adapted from previous studies, were used in the estimation of *R*_*0*_. These methods are nearest infectious neighbour, epidemic doubling time and compartmental susceptible-infectious (SI) method [20].

### Nearest infectious neighbour method

In this method, we used the GPS coordinates of affected herds to determine location and used the month when the first death was reported to determine its period of occurrence. To identify potential sources of infection for subsequent outbreaks, cases were ordered by month. Euclidean distances between the outbreak of interest and potential sources of infection were calculated using the spherical law of cosines [35]. The infection source for each outbreak of interest was identified as the outbreak that had occurred in the previous month and was closest by Euclidean distance. This was repeated until each herd was associated with an infection source [20]. A set (*S*), consisting of the number of outbreaks attributed to each infection source was constructed. A statistical technique, bootstrapping, was used to randomly select (with replacement) 1000 samples of size *n(S)* from *S*. The frequency distribution of sample means generated and the mean of this distribution was taken as the estimate of *R*_*0*_. Microsoft Excel 2010 was used to implement bootstrapping and to generate the mean distribution with the associated confidence interval (CI), within which we are 95% sure that the mean of the number of outbreaks attributed to each infection source lie. The CI was obtained by adding the margin of error (*d*) to the computed mean to obtain the upper bound (UB) and subtracting *d* from the mean for the lower bound (LB) where *d* is returned by software.

### Epidemic doubling time method

During the initial phase of an epidemic, the number of secondary cases increases exponentially, with each infection producing *R*_*0*_ new infections per generation assuming a constant doubling time (td) [20]. Anderson and May [21] defined a relationship between doubling time (td) and *R*_*0*_ as *R*_0_ = 1+(T / td)*log_*e*_2 where T is the herd infectious period. For the outbreaks studied here, the herd from which the first case of death was reported was considered the index case. Each herd that was subsequently infected was considered to present a new outbreak. Outbreaks were ordered by month and the average time for the number of outbreaks to double (td) for all possible combinations during this phase were computed using Microsoft Excel 2010. We assumed an infectious period of one month because data was aggregated at a monthly scale and as mentioned there is evidence from the literature that herds can remain infectious for a prolonged period [36]. We then used the doubling time and infectious period to estimate *R*_*0*_ from the above equation.

### SI modelling method

This method has been described and used in a number of studies [20,24,25,31]. We describe three approaches for estimating *R*_*0*_ using a simple deterministic SI model of the epidemic process. First we estimated the transmission rate, *β*, from epidemic data using a linear regression model (LRM) following an approach as described by Eblé et al. [37] and Gulenkin et al. [17]. The regression model was defined as *log* (−*log* (1−*E*(*C*) / *S*)) = *log* (*β*) + *log* (*I*Δ*t* / *N*), where *C*, *S*, *I* are respectively the number of newly-infected herds, susceptible herds and infectious herds at the start of the time interval Δt. We used Microsoft Excel 2010 to run the regression model. These estimates were bootstrapped and their mean taken as an estimate of *β*. *R*_*0*_ was then estimated from the product of *β* and T where T is the infectious period of the herd.

Secondly, a curve fitting (CF) approach was used to fit a Susceptible-Infectious-Removed (SIR) model to the epidemic data in order to estimate *β* and the removal rate *γ*. This approach was used as described by Gulenkin et al. [17]. Curve fitting was implemented using a modelling software package *Berkeley Madonna* ver. 8.3.18. These two parameters were then used to compute an estimate for *R*_*0*_ from (*S*_0_ * *β*) / *γ*, where *S*_*0*_ is the size of the susceptible population.

Lastly, we estimated *β* using an approach that describes disease transmission between epidemiological units in a Susceptible-Infectious (SI) model [20,24]. Here we assumed that all newly infected herds *(C)* were infected via contact with infectious herds *(I)* during the period of interest. Repeated infections reported from the same herd were considered to represent distinct outbreaks if they occurred in a period of more than two months of each other. Stegeman et al. [31] and Bett et al. [24] have shown that the number of new cases/outbreaks *C* is given by *βSI* / *N* from which *β* can be estimated given *N* as the total number of herds. The basic reproductive number *R*_*0*_ is calculated as the product *β*T, where T is the infectious period. Microsoft Excel 2010 was used to estimate the monthly *β* which was then analysed using bootstrapping techniques [24].

Sensitivity analysis was performed to assess whether the initial number of susceptible herds (*N*) had an effect on the estimate of *R*_*0*_, assuming *N* lies between (3 100–12 400) [38]. Due to the poor temporal resolution of the data arising from the reporting timescale, it was not possible to perform a sensitivity analysis of *R*_*0*_ to variation in the infectious period.

## Results

**Fig 1:**
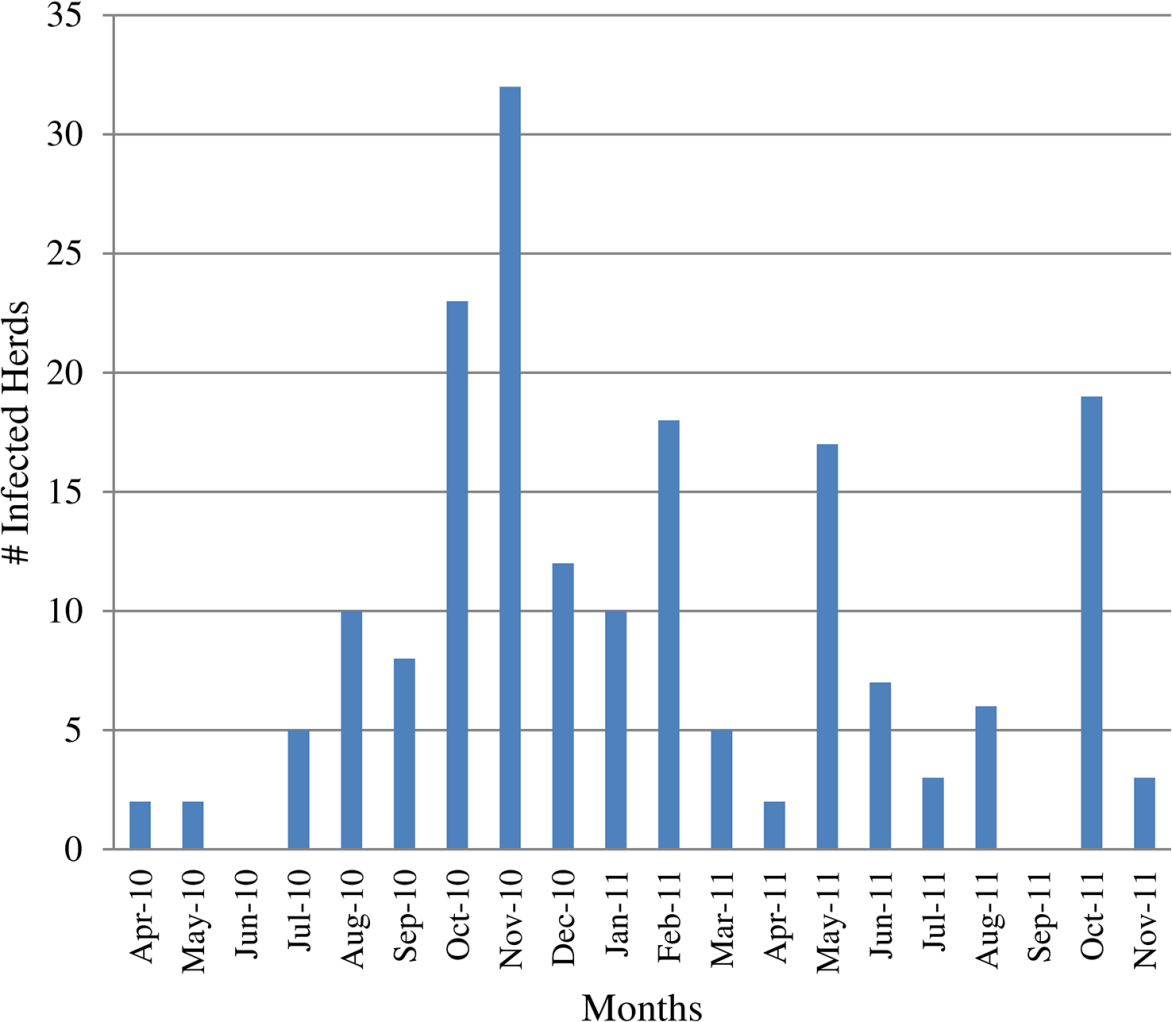
Number of African swine fever infected herds per month in Gulu District, Uganda, April 2010—November 2011.

During the study period, ASF resulted in a total of 1141 deaths in 211 herds in 43 villages in Gulu district. We present the distribution of infected herds per month in Fig 1. Table 1 summarises all the parameters obtained using each of the method.

**Table 1:** Summary of the parameters estimated using the different methods.

Method		Parameter estimates				Confidence Interval	
		*β*	td	γ	*R_0_*	LB	UB
Nearest infectious neighbour		-	-	-	3.24	3.21	3.27
Epidemic doubling time		-	1.106	-	1.63	1.56	1.72
SI model	LRM	1.90	-	-	1.90	1.87	1.94
	CF	0.0059	-	0.8236	1.58	-	-
	SI/N	1.77	-	-	1.77	1.74	1.81

### Estimate of *R*_*0*_ from the nearest infectious neighbour method

**Fig 2:**
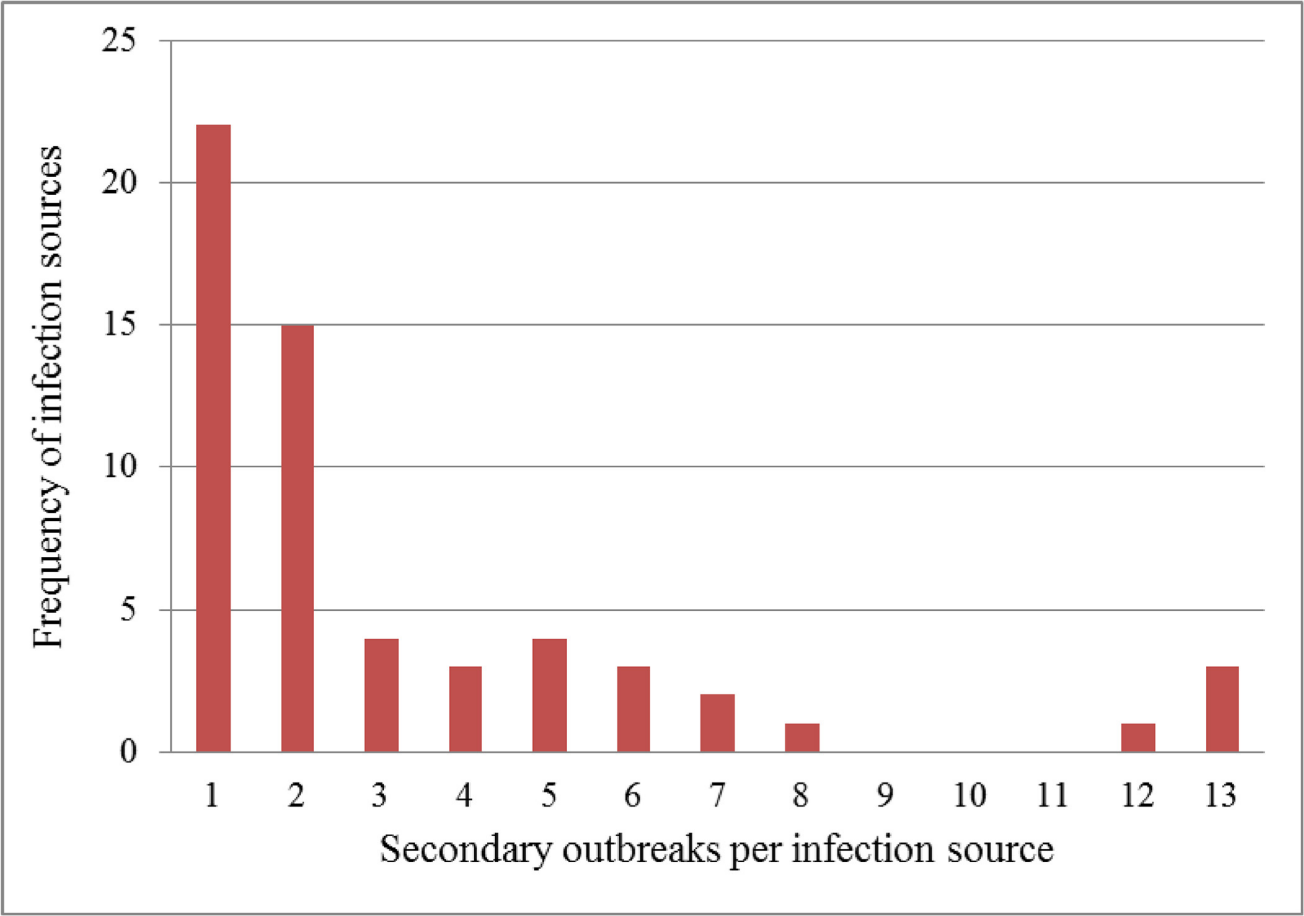
Distribution of secondary cases of African swine fever per infection source, Gulu District, April 2010—November 2011.

Based on our inclusion criteria, a total of 58 herds were identified to be sources of infection for at least one other herd. Fig 2 shows the distribution of source herds according to the number of secondary infections they presumably caused. S1 Fig shows the generation tree of ASF transmission following the nearest infectious neighbour route.

**Fig 3:**
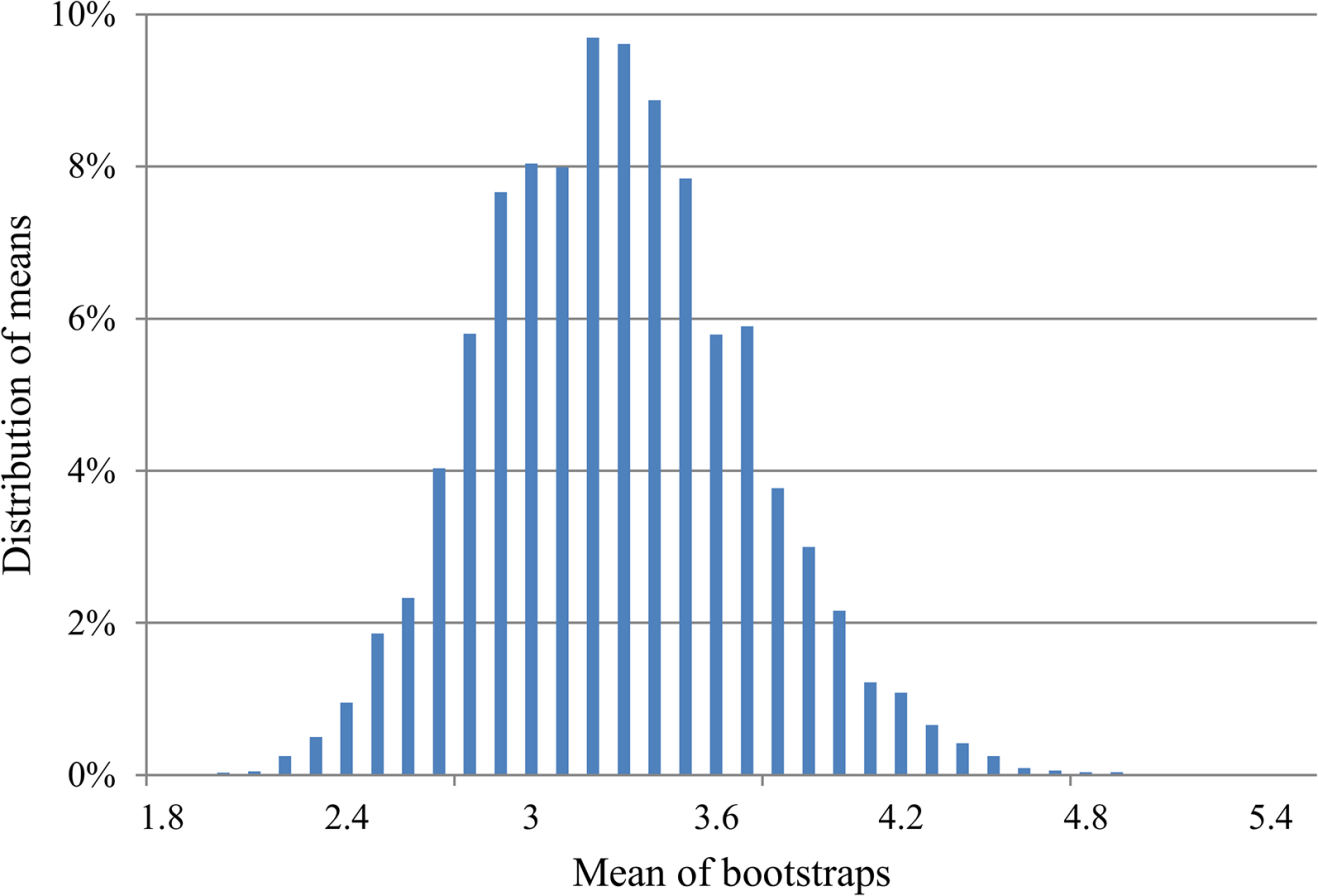
Distribution of bootstrapped number of secondary outbreaks per infection source.

The number of secondary infections per infection source ranged from one to 13 with 22 herds presumed to have caused only one secondary infection and three herds resulted in 13 secondary infections each. The number of secondary outbreaks was bootstrapped (n = 1000), generating the frequency distribution shown in Fig 3. The bootstrap distribution had an overall mean value of 3.24. This method therefore estimated *R*_*0*_ = 3.24 (95%CI: 3.21–3.27).

### Estimate of *R*_*0*_ from the epidemic doubling time method

**Fig 4:**
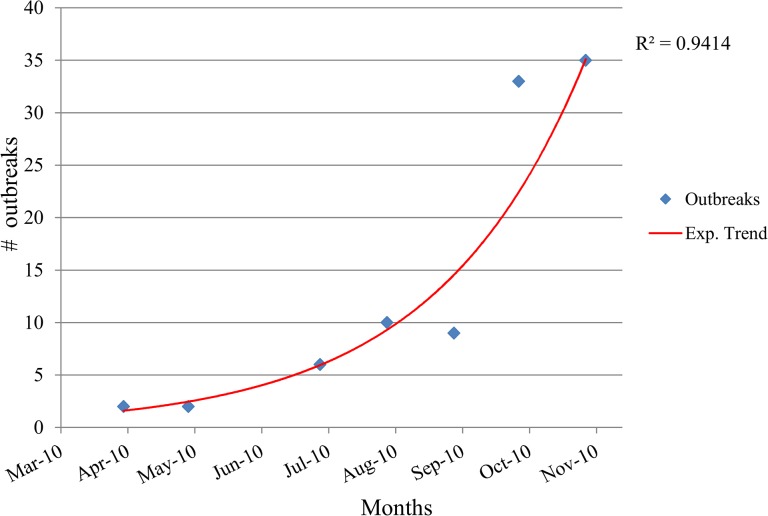
Exponential curve fitted to the data from the first phase (April—November 2010) of the African swine fever outbreaks in Gulu District, Uganda.

During the initial period of study (April—November 2010), the number of outbreaks increased exponentially as depicted in Fig 4. The computed average doubling time (td) during the initial phase was 1.106 (95%CI: 0.97–1.25) months. Using this doubling time, we estimated *R*_*0*_ to be 1.63 (95%CI: 1.56–1.72).

### Estimate of *R*_*0*_ from the SI modelling method

#### Linear regression approach

Using the linear regression model approach the estimate for *β* was 1.90 (95% CI: 1.87–1.94) herds per infected herd per month resulting in an *R*_*0*_ of 1.90 (95%CI: 1.87–1.94) since the infectious period is one month.

#### Curve fitting approach

Using the curve fitting approach an SI model was fitted to the epidemic data and the results are as shown in S2 Fig. Here *β* was estimated to be 0.0059 herds per infected herd per month while *γ* was 0.8236 per herd per month. We were not able to compute the confidence interval using this approach. Nonetheless these two parameters were used to estimate *R*_*0*_ = 1.58.

#### SI/N proportion approach

In Table 2, we show how the proportion SI/N, the number of newly infected households *(C)*, the number of infected households *(I)* and the transmission rate (*β*) varied during the period of study. The monthly *β*_*i*_ estimates were bootstrapped (S3 Fig) giving an overall *β* of 1.77 herds per infected herd per month and using this *β* value; *R*_*0*_ was estimated to be 1.77 (95%CI: 1.74–1.81). Monthly *R*_*0*_ estimates were found to be robust with regard to variation in the initial number of susceptible herds (S4 Fig).

**Table 2:** Estimated monthly SI/N, β and R_*0*_ during African Swine Fever outbreaks.

Month	# Herds	*C*	*I*	*SI/N*	*β(CN/SI)*	*R*_*0*_
Apr-10	6198	2	0	0	-	-
May-10	6196	2	2	2	1.00	1.00
Jul-10	6190	6	2	2	3.00	3.00
Aug-10	6180	10	6	6	1.67	1.67
Sep-10	6171	9	10	10	0.90	0.90
Oct-10	6138	33	9	9	3.70	3.70
Nov-10	6103	35	33	32	1.08	1.08
Dec-10	6091	12	35	34	0.35	0.35
Jan-11	6079	12	12	12	1.02	1.02
Feb-11	6061	18	12	12	1.53	1.53
Mar-11	6056	5	18	18	0.3	0.28
Apr-11	6054	2	5	5	0.41	0.41
May-11	6034	20	2	2	10.28	10.28
Jun-11	6027	7	20	19	0.36	0.36
Jul-11	6024	3	7	7	0.44	0.44
Aug-11	6016	8	3	3	2.75	2.75
Oct-11	5992	24	8	8	3.10	3.10
Nov-11	5989	3	24	23	0.13	0.13

## Discussion

In this study, three methods were used to estimate *R*_*0*_ from ASF epidemic data in a predominantly free-ranging pig production system in northern Uganda. The mean estimates for *R*_*0*_ ranged between 1.58 and 3.24. Considering the estimates from all the methods used, the nearest infectious neighbour method yielded the highest estimate when compared to estimates from doubling time and the SI model methods. This discrepancy could partly be a consequence of the assumptions made pertaining to the characteristics of the population system.

The assumed period of infectiousness of herds is plausible given the existing factors that may favour prolonged infectiousness specifically pig agistment, increased sales and home slaughtering of sick animals [16]. Increased survival of some of the shedding animals may also favour a prolonged infectious period, as does the likely survival of the pathogen, which is a highly stable DNA virus, in the environment outside its host [11,36].

Under-reporting of outbreaks has been reported to influence transmission parameters estimates specifically leading to underestimation of *R*_*0*_, yet in most epidemics, a significant fraction of outbreaks may go unreported [20]. However, for the purposes of this analysis, we assumed that all outbreaks during the study period were reported. This assumption is supported by the fact that, in the study area, farmers were primed to report outbreaks due to the ongoing research activities. There were frequent information dissemination exercises by the research team which we expect to have minimized the rate of reporting failures. In the event that some outbreaks were unreported, then our analyses may have underestimated *R*_*0*_.

Since *R*_*0*_ is known to be both population- and pathogen- specific [39] due to its sensitivity to production system, contact structure and environmental factors, it is interesting that our *R*_*0*_ estimates from the nearest infectious neighbour and doubling time methods are in close agreement with those of Gulenkin et al. [17] and Iglesias et al. [40] who estimated *R*_*0*_ to range from 2 to 3 and 1.58 respectively at the between-farm level. This could be just a matter of coincidence since, for example, estimation approaches that ignore the latent period of an infection tend to underestimate its *R*_*0*_ [41–43]. Therefore comparison of estimates from different studies and geographical areas should be made with caution. The true value of *R*_*0*_ for most epidemics may be difficult to quantify for a number of reasons. The source of each outbreak is usually unknown, reporting time-scales are frequently inconsistent and obtaining good contact tracing data is further complicated by the existence of multiple indirect routes of infection, farming systems and the role of human behaviour in transmission of ASFV and other pathogens [28]. Human behavioural factors such as poor handling and processing of pork and pork products at slaughter slabs, butchers and pork joints (i.e., makeshift kiosks where pork is roasted and eaten), farmers’ attitudes and cultural beliefs regarding handling of sick and dead animals, and use of swill are known risk factors for ASF transmission that may have influenced our estimates [16].

Gulenkin et al. [17] have identified road network density and pig density as significant risk factors for disease spread. The spatial distribution of ASF infected herds (April 2010—November 2011) shown in S5 Fig confirms that road network density and pig population density are key risk factors that may have also influenced our estimates. Their effect on our estimates needs to be investigated further and quantified. Despite these uncertainties, empirical data from epidemics can be a valuable source for estimating epidemiological parameters.

De Carvalho Ferreira et al. [11] assert that controlling an ASF outbreak is highly dependent on measures implemented by veterinary authorities, such as ‘stamping out’ (slaughter) of infected herds and quarantining affected areas. However, such measures are only feasible in countries which have economic means to compensate farmers. In resource constrained countries such as Uganda, the only feasible measures focus on preventive mitigation, including enhanced biosecurity, and early detection and response. Estimates of *R*_*0*_ can inform the efficient application of these measures.

Generally, few if any attempts have been made to estimate *R*_*0*_ from field data in the endemic regions of Africa. Here we have estimated *R*_*0*_ for ASF in a predominantly free-ranging production system, a system that is common in many parts of East Africa. All the mean estimates were above one which is consistent with the observed persistence of disease in the population. This is indicative of the inadequacy of the existing control measures in curbing ASF dissemination thereby requiring enhanced effort in devising new strategies or improving adherence to existing ones. In conclusion, we recommend that more epidemiological studies be designed to collect daily outbreak data from the field this will enable the relaxation of several assumptions made in this work and result in more accurate estimates of *R*_*0*_.

## Supporting Information

S1 Data The data that was used in all the computations and Figures. (ZIP)

S1 Fig Generation tree following the nearest infectious neighbour route. Nearest infectious neighbour generation tree also known as a transmission network. Epidemic is suspected to have been introduced at herd/ node 1 coloured red (bottom extreme left). The critical node at which the disease could have been stopped from further spread as highlighted in green in the generation tree. (Designed in network analysis tool ORA)
(TIFF)

S2 Fig The SIR model used to simulate outbreak data of African swine fever, Gulu District, Uganda, April 2010—November 2011. (TIFF)

S3 Fig Distribution of bootstrapped monthly transmission rate coefficient *β* estimates. (TIFF)

S4 Fig Sensitivity of basic reproduction number *R*_*0*_ to variation in initial number of herds. (TIFF)

S5 Fig Spatial distribution of ASF infected herds (April 2010—November 2011). (TIFF)

S1 Text Philosophical underpinning of *R*_*0*_. (PDF)
