# Supplementary figures and images for: Estimating the Basic Reproductive Number (R0) for African Swine Fever Virus (ASFV) Transmission between Pig Herds in Uganda
Source: PLoS One. 2015 May 4;10(5):e0125842. doi: 10.1371/journal.pone.0125842 (PMC4418717; doi:10.1371/journal.pone.0125842)

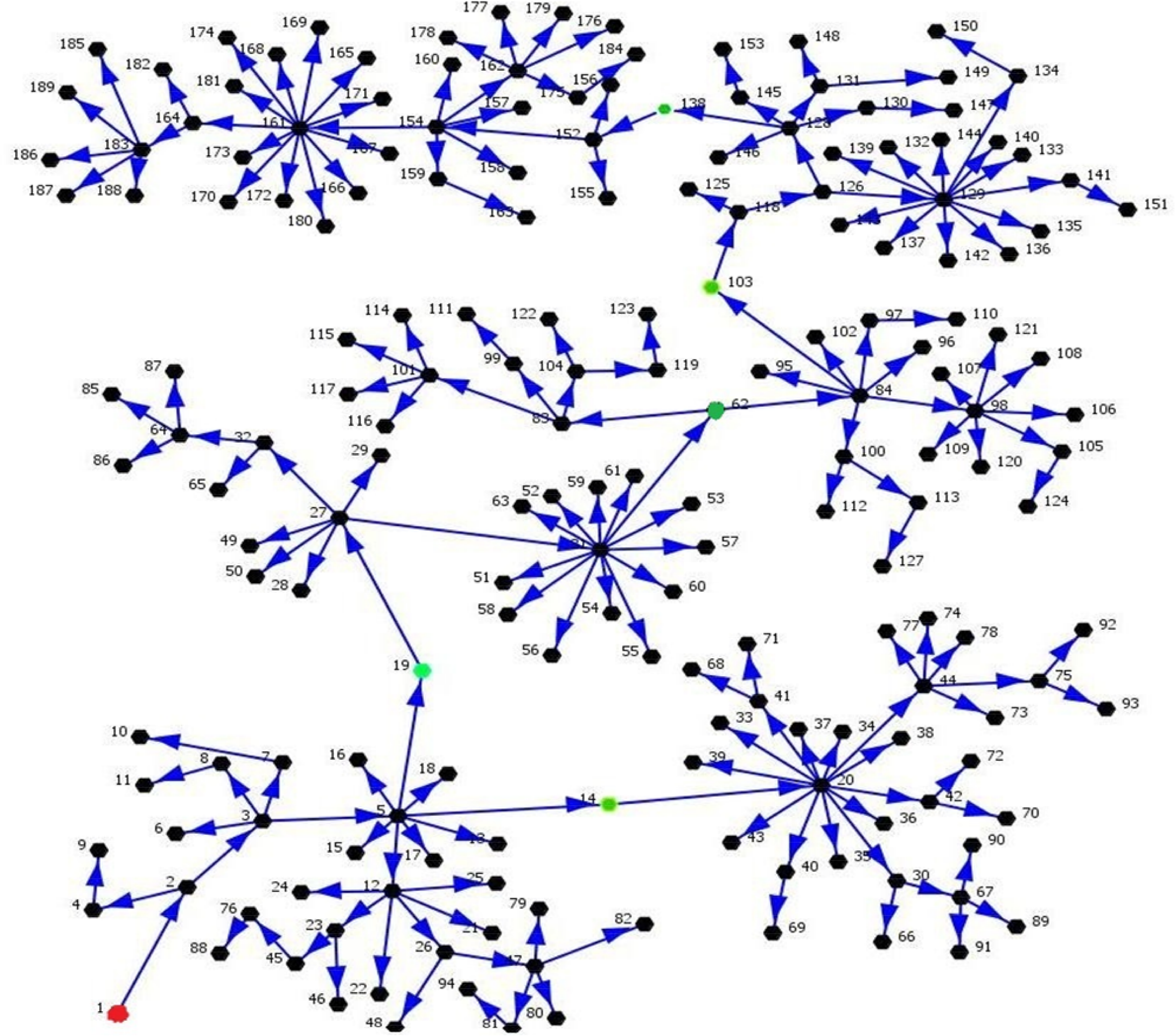

Supplement: S1 Fig — Nearest infectious neighbour generation tree also known as a transmission network. Epidemic is suspected to have been introduced at herd/ node 1 coloured red (bottom extreme left). The critical node at which the disease could have been stopped from further spread as highlighted in green in the generation tree. (Designed in network analysis tool ORA) (TIFF) [file pone.0125842.s002.tiff]

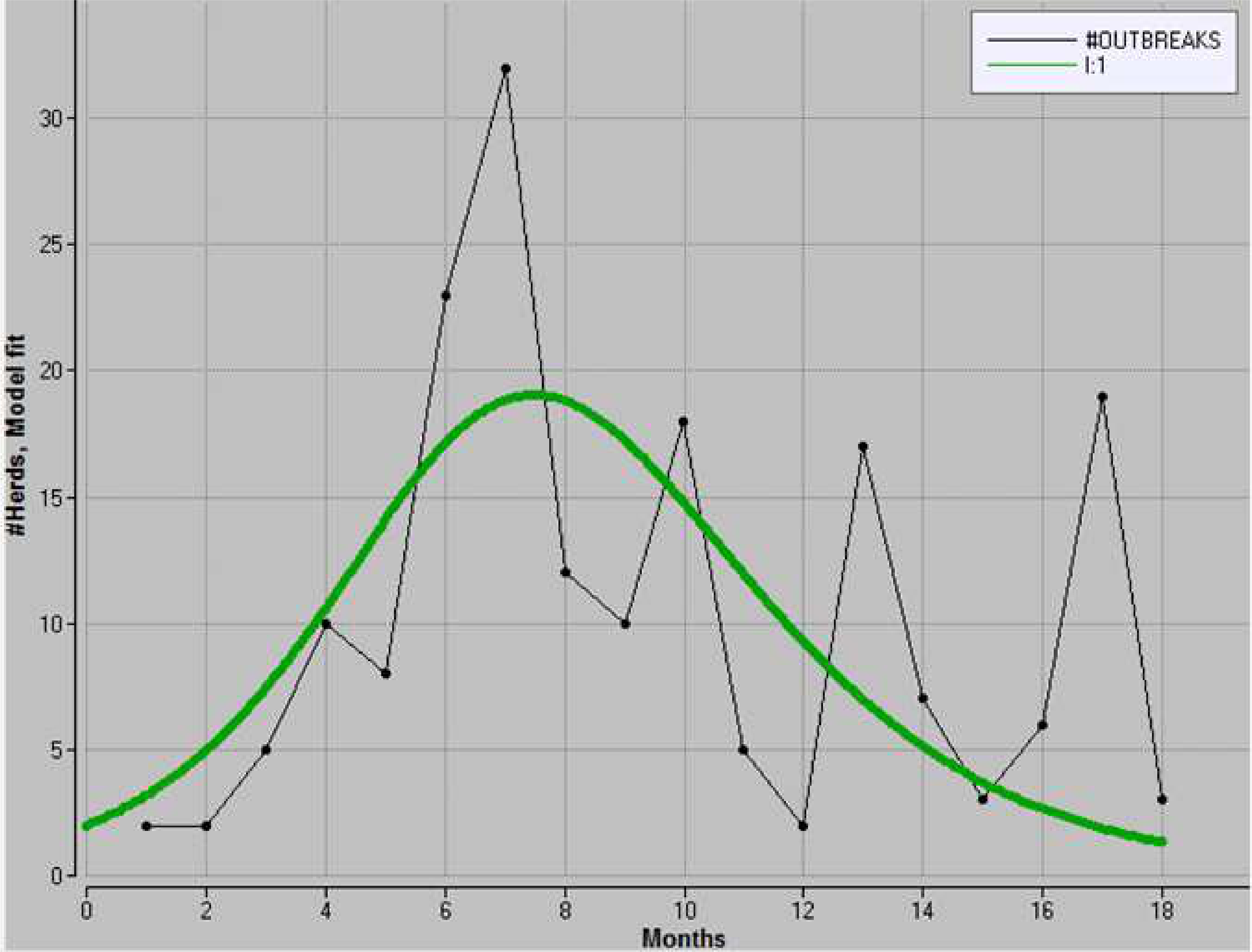

Supplement: S2 Fig — (TIFF) [file pone.0125842.s003.tiff]

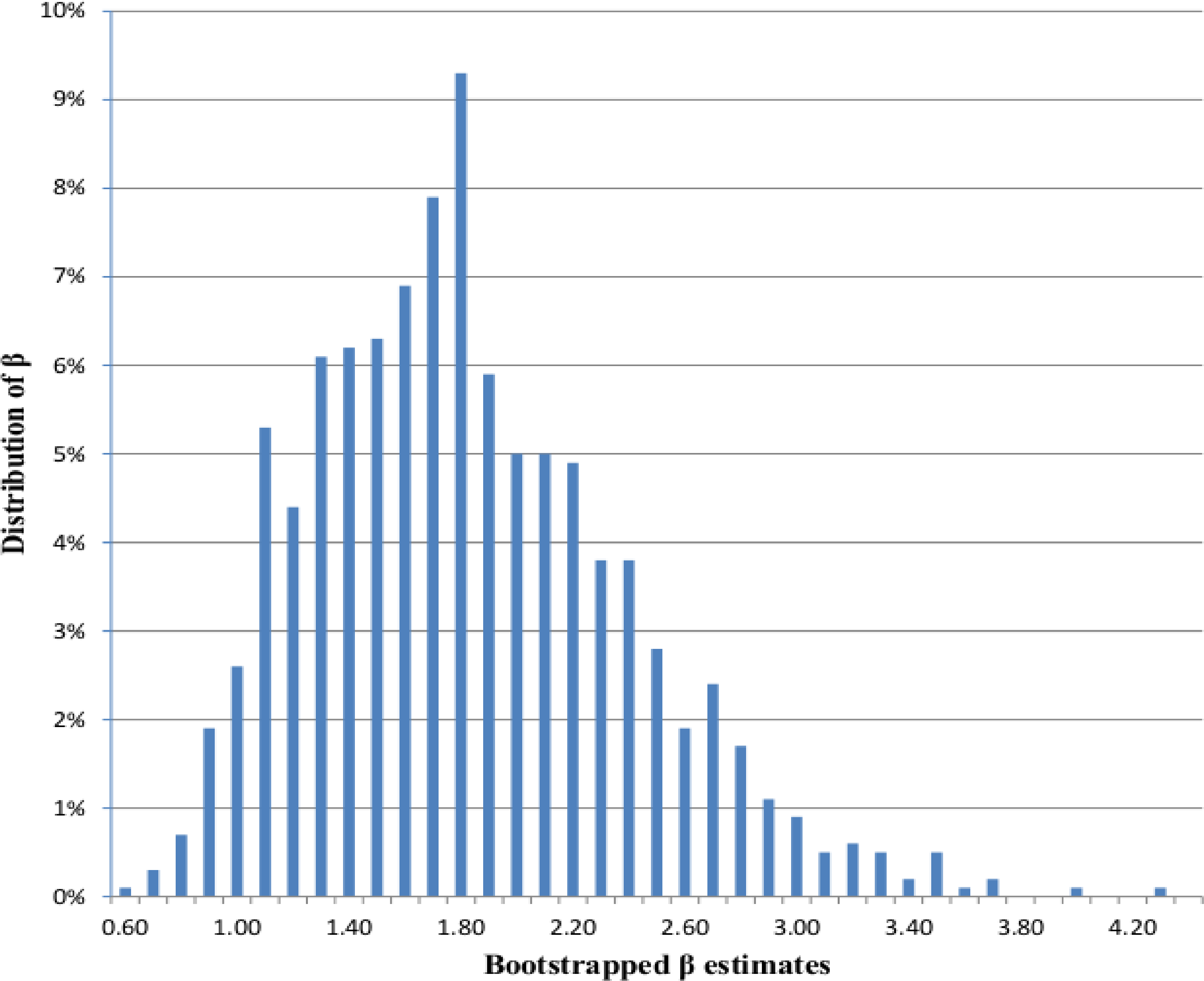

Supplement: S3 Fig — (TIFF) [file pone.0125842.s004.tiff]

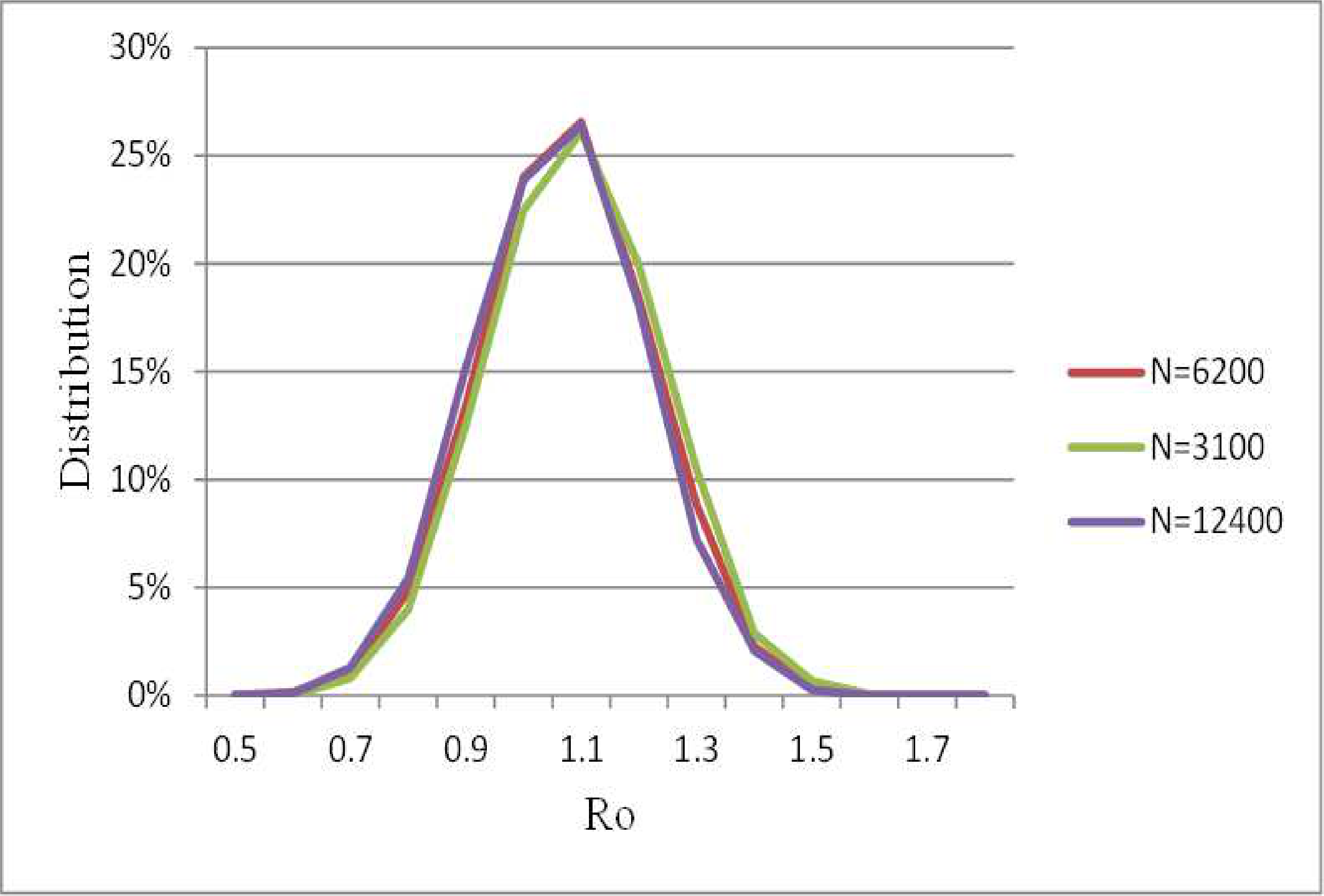

Supplement: S4 Fig — (TIFF) [file pone.0125842.s005.tiff]

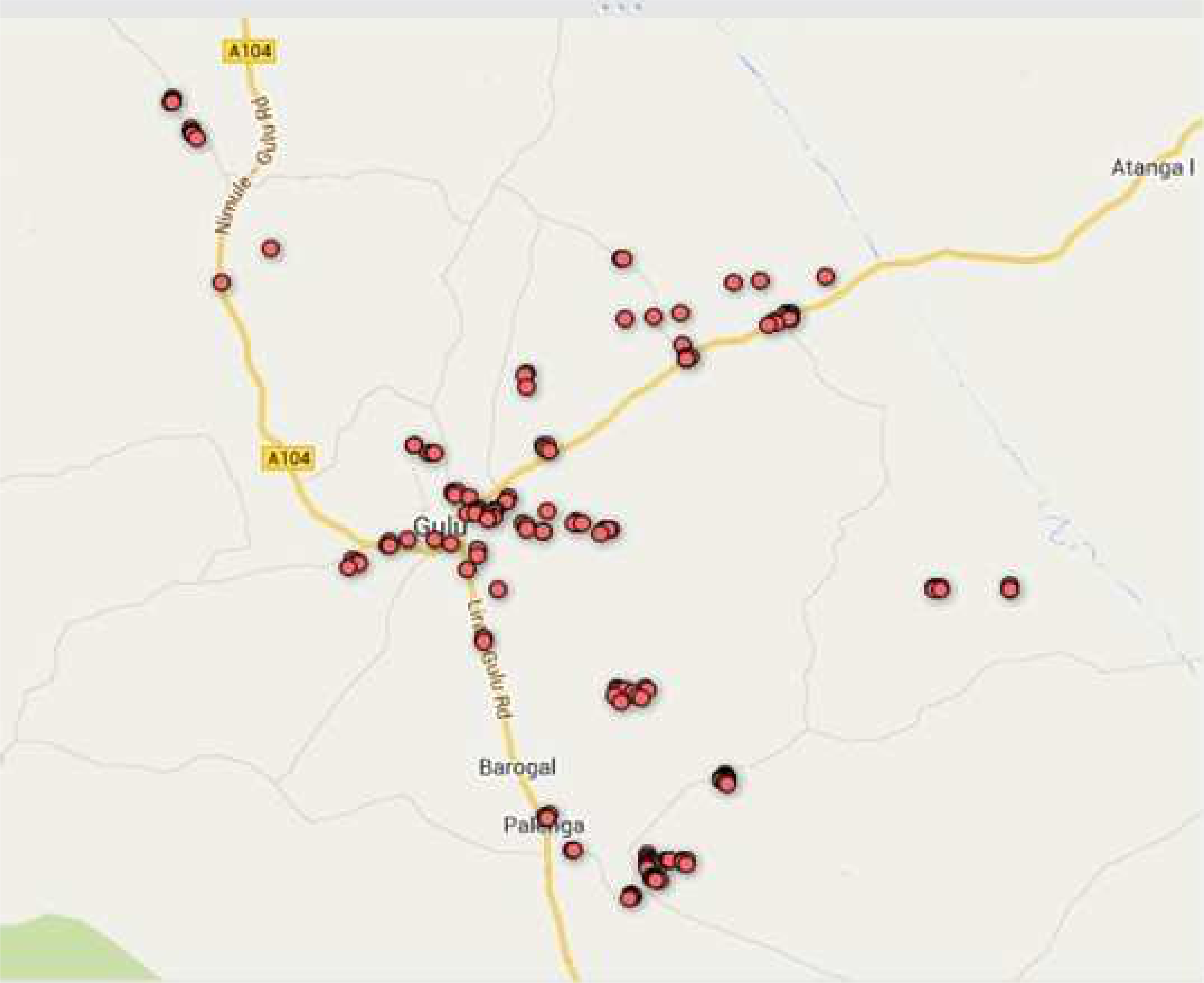

Supplement: S5 Fig — (TIFF) [file pone.0125842.s006.tiff]
