# Supplementary material for: Estimating the Basic Reproductive Number (R0) for African Swine Fever Virus (ASFV) Transmission between Pig Herds in Uganda
Source: PLoS One. 2015 May 4;10(5):e0125842. doi: 10.1371/journal.pone.0125842 (PMC4418717; doi:10.1371/journal.pone.0125842)
Supplement: S1 Text — (PDF) [file pone.0125842.s007.pdf]

Philosophical underpinning of R_0

Consider a 'closed' and 'naive' pig population that is in one way or the other invaded by a disease-causing organism. For simplicity of derivations, the following assumptions are made:

- There is homogeneous mixing of individuals
- That all subsequent infections are a result of contacts between susceptible (S) and the infectious (I) individuals
- That every infection results into either death or immunity (R)

Under these assumptions, the following state-transition SIR (density-dependent) model is formulated:

$$\frac{dS}{dt} = -\beta SI, \qquad (1)$$

$$\frac{dI}{dt} = \beta SI - \gamma I, \qquad (2)$$

$$\frac{dR}{dt} = \gamma I. \qquad (3)$$

Subject to the initial conditions: $S(0) = S_0 > 0$; $I(0) = I_0 > 0$ and $R(0) = 0$. (4)

Where β is the transmission rate (a product of probability of transmission and contact rate) and is the removal rate ($1/\gamma$ = average infectious period). Give the initial conditions and parameters, we are interested in knowing if the infection will spread or not and how it will develop over time. Initially, it follows from equation (2), that:

$$\frac{dI}{dt} = (\beta S_0 - \gamma)\, I_0$$

If $\frac{dI}{dt} > 0$, $(\beta S_0 - \gamma) > 0$, then I(t) starts to increase and there is likelihood of an epidemic. This implies $\beta S_0 > \gamma$ or $\beta S_0 / \gamma > 1$. The expression $\beta S_0 / \gamma$ is the reproduction number (*R_0*) of the infection. If $\frac{dI}{dt} \leq 0$, then $(\beta S_0 - \gamma) \leq 0$, then I(t) remains below I_0 implying the epidemic does not occur. If $R_0 < 1$, the disease dies out, whereas if $R_0 \geq 1$, the disease persists in the population (Li et al., 2011).
